# Spinal hypermobility accelerates ossification in posterior longitudinal ligaments: insights from an *in vivo* mouse model

**DOI:** 10.3389/fphys.2025.1561199

**Published:** 2025-03-19

**Authors:** Tao Tang, Zhengya Zhu, Zhongyuan He, Fuan Wang, Lin Chen, Jianfeng Li, Hongkun Chen, Jiaxiang Zhou, Jianmin Wang, Shaoyu Liu, Yunfeng Yao, Xizhe Liu, Zhiyu Zhou

**Affiliations:** ^1^ Department of Orthopedic Surgery, The Second Affiliated Hospital of Anhui Medical University, Hefei, Anhui, China; ^2^Innovation Platform of Regeneration and Repair of Spinal Cord and Nerve injury, Department of Orthopaedic Surgery, The Seventh Affiliated Hospital, Sun Yat-sen University, Shenzhen, China; ^3^ Department of Orthopaedics, Affiliated Hospital of Xuzhou Medical University, Xuzhou, China; ^4^ Department of Orthopaedics, The Second Affiliated Hospital of Chongqing Medical University, Chongqing, China; ^5^ Department of Ophthalmology, The Second Affiliated Hospital of Anhui Medical University, Hefei, China; ^6^ Department of Orthopaedic Surgery, The Affiliated Hospital of Qingdao University, Qingdao, China; ^7^ Guangdong Provincial Key Laboratory of Orthopedics and Traumatology, The First Affiliated Hospital of Sun Yat-sen University, Guangzhou, China

**Keywords:** OPLL, heterotopic ossification, spinal hypermobility, animal models, spinal cord compression

## Abstract

**Introduction:**

Ossification of the posterior longitudinal ligaments (OPLL) is characterized by heterotopic ossification in the posterior longitudinal ligament of spine. Our earlier research found that mechanical stimulation enhances osteogenic differentiation in OPLL-derived ligament cells. Nevertheless, the function of hypermobility of the spine on ligament ossification remain unexplored *in vivo*.

**Methods:**

We created the novel stimulation device to induce spinal hypermobility in mice with heterotopic ossification of the spine ligaments. The mice were randomly divided into three groups, control, slow hypermobility (SH) group and fast hypermobility (FH) group according to the frequency of spinal movement. Ligament ossification and changes in spinal range of motion (ROM) were assessed using micro-CT and X-rays. Morphological alterations were examined through HE staining. Behavioral evaluation was performed using the Basso Mouse Scale (BMS) score and inclined plane test (IPT). Immunofluorescence was employed to examine the expression of related proteins.

**Results:**

After 8 weeks, it showed increased ligament ossification and chondrocyte proliferation both in SH and FH group. After 16 weeks, The BMS score and IPT were lower both in the SH and FH group compared to the controls. Additionally, the ROM of cervicothoracic and thoracolumbar spine was lower in the FH group than in the controls. Immunofluorescence analysis revealed increased levels of SP7, RUNX2, OCN, DLX5, NOTCH1, and HES1 in the ligament tissues of the FH group compared to controls.

**Conclusion:**

spinal hypermobility promotes the progression of ossification in mice with heterotopic ossification of the spine, shedding new light on the pathogenesis of OPLL.

## 1 Introduction

Ossification of the posterior longitudinal ligaments (OPLL) is characterized by ectopic bone formation in the posterior longitudinal ligament (PLL) of the spine (Inamasu et al., 2006). In many cases, OPLL is accompanied by spinal cord and nerve root compression, which may lead to severe limb paralysis and motor impairment, causing a serious financial strain to families and society (Boody et al., 2019). Surgery is the primary form of therapy for severe OPLL, however, the etiology of OPLL is currently incompletely understood (Bernstein et al., 2019).

OPLL is considered a multifactorial disease, with age, diabetes, obesity, diet, mechanical factors and femoral neck bone density associated with the development of OPLL (Kobashi et al., 2004; Yoshimura et al., 2014; Chen X. et al., 2016; Liu et al., 2022). Clinically, recurrent ossification following posterior decompression in OPLL patients is partly attributable spinal instability produced by damaging posterior spinal structure (Nishida et al., 2017). Cyclic stretch stimulation promotes osteogenic differentiation of OPLL-derived ligament cells (Iwasawa et al., 2006; Chen D. et al., 2016; Sugita et al., 2020). Our prior research shown that cyclic stretch enhanced osteogenic development of OPLL ligament cells by activating the DLX5 and YAP-Wnt/-catenin axis (Zhu et al., 2023b). The investigation of the role and underlying mechanisms of mechanical factors in OPLL remains unknown *in vivo*.

The regulation of mineralization in soft and skeletal tissues is a well-known function of ENPP1. It mainly performs its role by producing pyrophosphate, which is an essential inhibitor of the production of hydroxyapatite and its deletion is suitable for animal model for studying OPLL (Okawa et al., 1998; Roberts et al., 2019; Cheng et al., 2021). Male mice with *Enpp1* gene deletion exhibits spontaneous heterotopic ossification in posterior longitudinal ligaments in this study. We used spinal hypermobility equipment to identify effect of mechanical stimulation on progression of PLL ossification in *Enpp1*-deficient mice. In this work, the mice undergo repeated neck flexion and extension movements using spinal hypermobility device. It was shown that excessive spinal movement accelerated the development of ligament ossification and spinal range of motion and motor function were affected based on the analysis of micro-CT and relevant scoring results. This work examines the significance of hypermobility in OPLL and investigates the molecular mechanism of OPLL development.

## 2 Materials and methods

### 2.1 Animals

Construction of the *Enpp1*-deficient mice was performed as previously described (He et al., 2024). The transgenic mice used in this study were raised indoors and provided with a normal laboratory diet. All behavioral experiments were performed during the same circadian cycle (with natural light from 8:00 a.m. to 8:00 p.m.). This work was authorized by the Sun Yat-sen University Ethics Committee for Animal Experimentation (No. 2023000404). All animal experiments should comply with the ARRIVE guidelines and should be carried out in accordance with the National Research Council’s Guide for the Care and Use of Laboratory Animals. All procedures were carried out in accordance with the laboratory animal center’s animal care requirements.

### 2.2 Spinal hypermobility equipment

**FIGURE 1:**
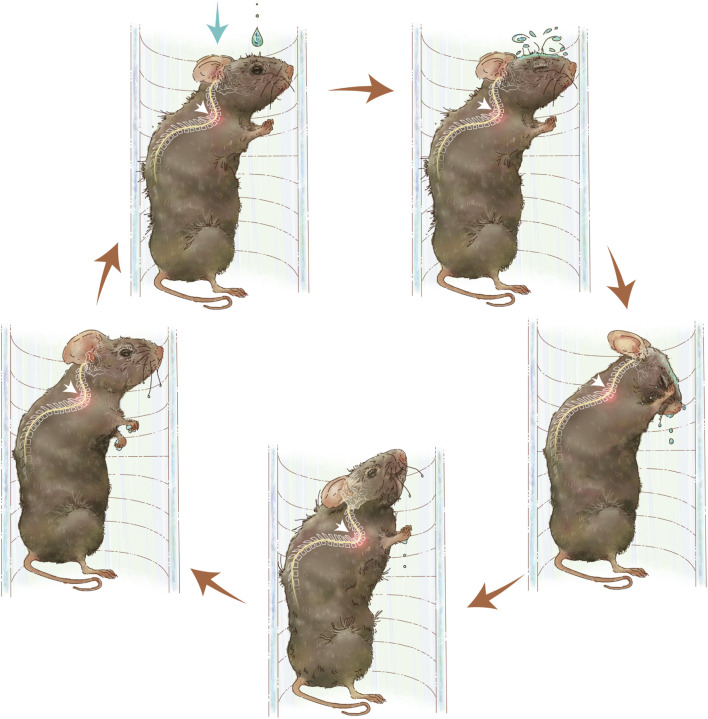
Diagram of spinal hypermobility injury pattern. Repeated neck flexion and extension motions is caused by water droplets on the skin of the mice. The activity of the cervicothoracic spine following hypermobility is shown by white arrows.

**FIGURE 2:**
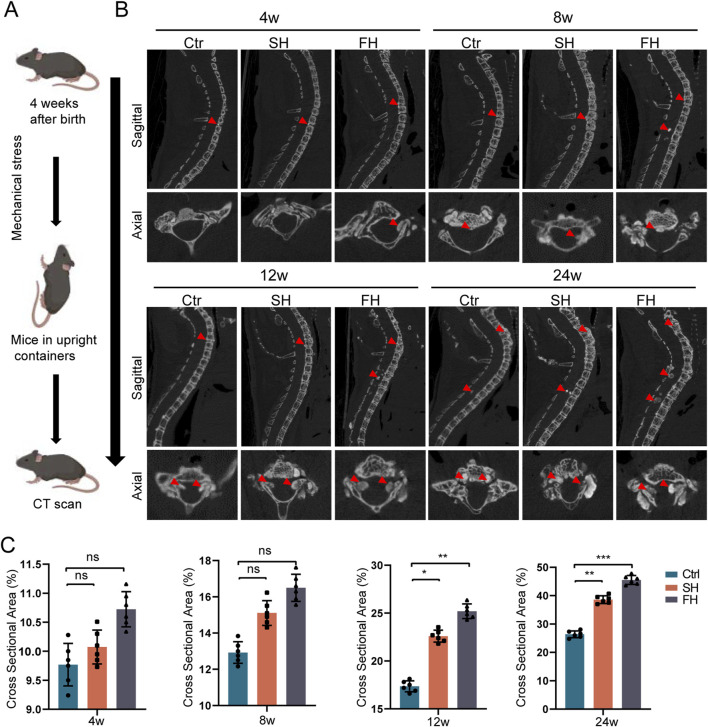
*Enpp1*-deficient mice showed a significant increase in ossification of PLL after 8 weeks of hypermobility. **(A, B)** micro-CT sagittal and axial images of heterotopic ossification, n = 6, red arrows represent ossification formation in different regions. **(C)** Quantitative analysis of the relative cross-sectional area of bone masses in the cervicothoracic spinal canal of mice, n = 6, all data are expressed as mean ± SD, *p < 0.05, **p < 0.01, ***p < 0.001.

Seventy-two *Enpp1*-deficient male mice were randomly divided into three groups, control, slow hypermobility (SH) group and fast hypermobility (FH) group. Each group had four time points with six mice per time point. The device consisting of a water dripper and a flow valve to induce repeated neck flexion and extension motions in mice. Postnatal 4-week-old mice were exposed to drip stimulation, while the control group remained untreated (Figure 1, Supplementary Video S1). The hypermobility was administered using drip stimulation 4 weeks after birth, while the control group received no stimulation (Figure 2A). The dropper was suspended at the mouse’s upper and connected to a volumetric flask, and the spinal activity frequency of the mice was recorded at 45 times/min for SH group or 90 times/min for FH group by adjusting flow rate valve. The protocol was 60 min once a day, and 5 days per week. The duration of stimulation for each group was 4 weeks, 8 weeks, 12 weeks, and 24 weeks.

### 2.3 Range of motion of the spine

Real-time radiographs of mice in an active condition were taken (Dandong Science and Technology Instrument Co., LTD.). The Cobb angle, which is determined from the locations of the vertebrae that are most inclined above and below the curvature’s peak was used to calculate the curvature’s magnitude on radiographs (Kubota et al., 2013). This angle is generated by the intersection of two lines drawn at the curve’s end vertebrae. One line runs parallel to the superior endplate of the vertebrae, while the other runs parallel to the inferior endplate of the vertebrae (Morini et al., 2016). To indicate the change in spinal range of motion (ROM), we assessed the difference between the largest and smallest cobb angles in the cervicothoracic and thoracolumbar segments of mice during locomotion.

### 2.4 Micro-computed tomography

Spinal ligament ossification was evaluated in mice by using micro-CT (SCANCO MEDICAL, μCT100, Bassersdorf, Zurich, Switzerland). The scan parameters were as follows: tube diameter 72 mm, field of view/FOV 75 mm, voltage 70 KVp, current 20 μA, resolution 24.5 μm, integration time 270 ms, and height 105 mm. The 3D reconstruction of CT images of mice was performed using DateViewer software, and the image format was converted by DICOM converter and quantitative analysis of the relative cross-sectional area of heterotopic ossification was performed by BeeViewer software. To quantitatively analyze the ratio of ossification area within the cervicothoracic spinal canal to the total spinal canal area at different segments, we used the relative cross-sectional area of bone masses to measure the progression of ossification. Relative cross-sectional area of bone masses was equal to the maximum ossified cross-sectional area/total cross-sectional area within the vertebral canal of the corresponding segment.

### 2.5 Histological staining

*Enpp1-deficient* mice were euthanized by anesthesia overdose for histological analysis and the spine was dissected and fixed in 4% paraformaldehyde for 24 h and washed 3 times with PBS. The specimens were decalcified with EDTA, dehydrated in 20% solution, embedded with OCT. Frozen section sliced into 10 μm thick sections and stained with hematoxylin and eosin (H&E).

### 2.6 Immunofluorescence staining

We performed immunofluorescence staining on frozen sections as described below: PLL tissues were permeabilized and blocked in TBST (Biosharp, China) containing 0.3% Triton X-100 (Sigma, America) and 5% bovine serum albumin (BSA, BioFroxx, Germany). Sections were incubated with primary antibody overnight at 4 °C and goat anti-rabbit secondary antibody for 1 h at room temperature. Nuclei were counterstained with 4,6-diamidino-2-phenylindole (DAPI, Abcam) for 5 min. Stains were visualized using a fluorescence microscope (Leica, Germany). The following primary antibodies were used: rabbit monoclonal anti-DLX5 antibody (Abcam, 1:1000), rabbit monoclonal anti-Notch1 antibody (Abcam, 1:150), rabbit monoclonal anti-HES1 antibody (Abcam, 1:100), rabbit monoclonal anti-RUNX2 antibody (Abcam, 1:6400), rabbit monoclonal anti-SP7 antibody (Abcam, 1:1000), rabbit monoclonal anti-OCN antibody (Abcam, 1:100) and goat anti-rabbit secondary antibody (Abcam, 1:300).

### 2.7 Motor function evaluation

To evaluate the motor function after spinal hypermobility in *Enpp1-deficient* mice, the Basso Mouse Scale (BMS) scores for locomotion and an inclined plane test (IPT) were performed every 4 weeks. The BMS score is obtained by placing the mice in an empty cage and giving them a light stimulus to crawl and observing their hip, knee and ankle joint walking, trunk movements and their coordination. Mice were positioned horizontally on a smooth tilt board for the IPT. The board was originally oriented horizontally 0°, then the angle was adjusted by 10° after each try. The angle at which the mouse stayed on the board for 5 s was measured.

The ossification of the cervicothoracic spine was examined using the Mata score, which was graded on a scale of 0–4 based on the degree of ossification at each disc space level according to previous research (Mata et al., 1998). A score of zero indicates no ossification, one indicates ossification without bridging, two indicates partial bridging, three indicates full bridging of the intervertebral disc space, and four indicates severe ossification. The degree of ossification of the 2nd cervical to 4th thoracic vertebrae was scored at each intervertebral disc space level with a maximum score of 32.

### 2.8 Statistical analysis

SPSS version 20.0 (SPSS, United States) was used for statistical analysis. All values are presented as means ± standard deviation (SD). The Shapiro–Wilk normality test was performed to evaluate the normality of the data distribution. Statistical significance (*p* ≤ 0.05) was analyzed by Student’s t-test (two groups) or one-way analysis of variance (ANOVA) (more than two groups). Mann-Whitney U test was performed for non-normally distribution data.

## 3 Results

### 3.1 Heterotopic ossification of the ligament

We found that the spinal canal of C5-T12 segment was prone to ossification after drip stimulation through micro-CT, and we only counted the spinal ossification area at this level. After 8 weeks of stimulation, the hypermobility group exhibited increased ossification of the PLL and spinal canal. Following 12 weeks and 24 weeks of stimulation, it showed the ossification of spinal canal and PLL, with the FH group displaying the most extensive changes (Figures 2A, B). Furthermore, at 8, 12, and 24 weeks of stimulation, the relative area of ossification in the spinal canal was consistently larger in the hypermobility group compared to the controls, with the FH group showing the most significant increase (Figure 2C). In summary, starting from 8 weeks of stimulation, there was a progressive increase in the ossification level of the spinal canal and PLL in mice.

### 3.2 Weight change analysis and HE staining

**FIGURE 3:**
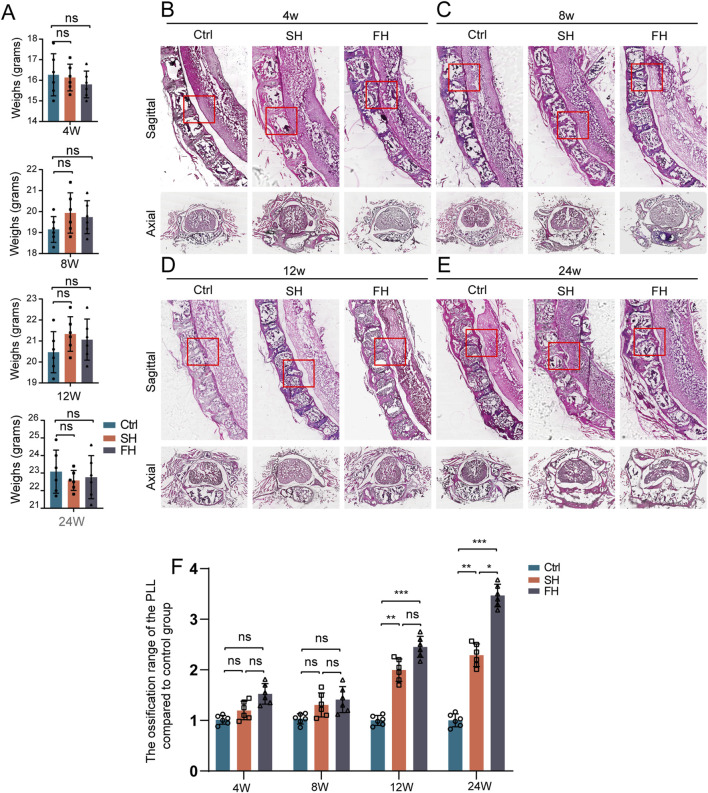
Weight changes and HE staining which subjected to spinal hypermobility. **(A)** The changes in body weight of mice were assessed under varied stimulation periods in the control group, slow hypermobility (SH) group and fast hypermobility (FH) group, all data are expressed as mean ± SD. **(B–E)** After stimulation for 4, 8, 12, and 24 weeks, images of the sagittal and axial mouse spine stained with HE is shown, with red boxes representing the production of heterotopic ossification. **(F)** Quantitative analysis was performed to assess the ossification range of PLL in the mobility group compared to the control group using HE staining. n = 6, *p < 0.05, **p < 0.01, ***p < 0.001.

First, we investigated whether spinal hypermobility had an impact on mouse body weight and we found no statistically significant difference in the various groups of body weight (Figure 3A). Then, HE staining was employed to compare the size of OPLL between different groups which was similar to micro-CT (Figures 3B–E). After 4 and 8 weeks of stimulation, the area of ossification increased slightly the mobility group compared to the control group, but the difference was not statistically significant. The area of PLL ossification increased significantly after 8 and 12 weeks of stimulation, however, there was no significant difference between SH group and FH group. Following 24 weeks of stimulation, the mobility group displayed spinal cord compression, with the compression of the spinal cord being the most visible in the FH group (Figure 3F).

### 3.3 Motor function scores

**FIGURE 4:**
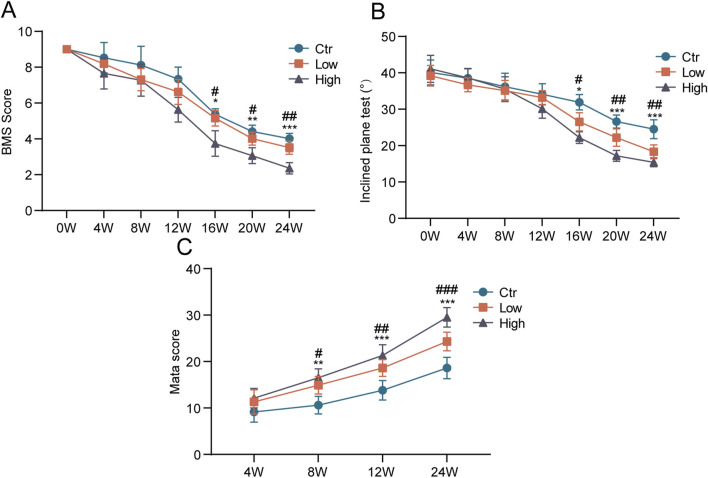
Effects of hypermobility on motor function in mice with heterotopic ossification of ligaments. **(A, B)** At the age of 4 weeks, exogenous stimulation was started. In the open field, mice were put to the test every 4 weeks. **(C)** Assessment of the effect of hypermobility on the degree of cervicothoracic ossification in mice based on the Mata scoring system, n = 6, all data are expressed as mean ± SD, # and * represent statistical differences between SH and FH groups, respectively, #*p* < 0.05, ##*p* < 0.01, ###*p* < 0.001, *p < 0.05, **p < 0.01, ***p < 0.001.

Using motor function scores, we evaluated the effect of hypermobility of spine on motor function. After 16 weeks of stimulation, the BMS score and maximum angle of stay on the inclined plate for 5 s were considerably lower in the hypermobility group than in the controls, with the high group being the most significant (Figures 4A, B). In addition, we evaluated the degree of ossification of the mouse spinal ligaments using micro-CT scans. The results showed that the cervicothoracic ossification scores increased over time in all groups according to the Mata scale, and the hypermobility group was significantly higher than the controls after 8 weeks of stimulation, with the highest ossification scores in the FH group (Figure 4C).

### 3.4 Spinal range of motion

**FIGURE 5:**
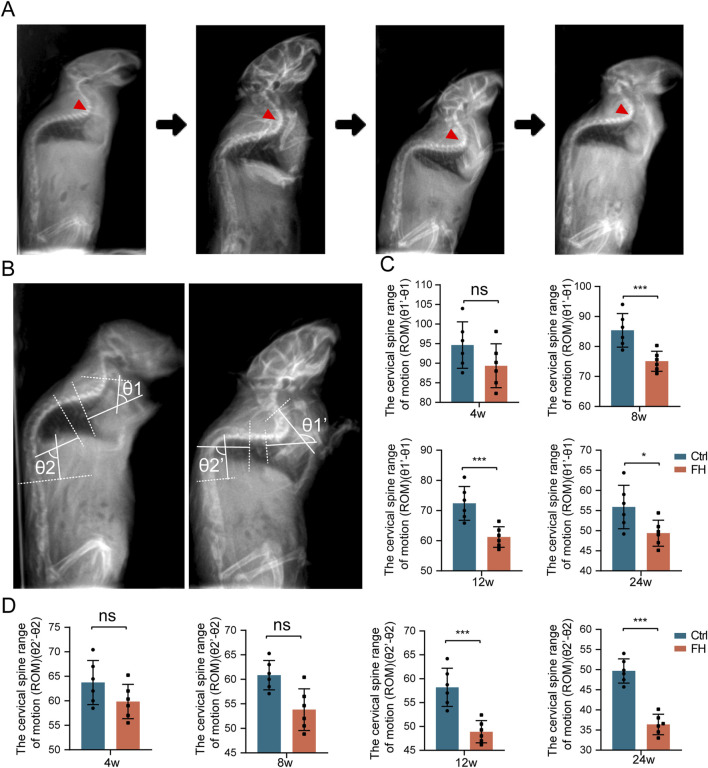
The ROM of the spine is altered during hypermobility. **(A)** Sagittal spine radiographs of mice before and after hypermobility. **(B)** Measurement of spinal ROM in the mice cervicothoracic and thoracolumbar regions. **(C, D)** The change in ROM in the cervicothoracic is represented by θ1′-θ1, while the change in ROM in the thoracolumbar is represented by θ2′-θ2, n = 6, all data are expressed as mean ± SD, *p < 0.05, **p < 0.01, ***p < 0.001.

In order to measure the spinal range of motion (ROM), we took X-radiography in the active state of the spine to capture motion trajectory of the mice (Figure 5A). The range of motion of the cervicothoracic and lower thoracic spine were larger in upright mice. To provide clarity, we have named the Cobb angle in the cervicothoracic segment (C5-T4) the θ1 angle and in the lower thoracic segment (T6-T12) the θ2 angle. We have previously confirmed that the FH group has a greater impact on spinal ossification and motor ability than SH group, and then we choose the FH group for further study. We investigated the impact of hypermobility on spinal ROM in mice by quantifying the difference between the maximum and minimum Cobb angles in the cervicothoracic and thoracolumbar segments (Figure 5B). There was no significant change in the ROM of cervicothoracic and thoracolumbar regions of the spine after 4 weeks of stimulation between the hypermobility group and controls. After 8 weeks, the cervicothoracic of ROM in the FH group was lower than in the controls, whereas there was no significant difference in thoracolumbar of ROM. After 12 weeks of stimulation, the FH group’s thoracolumbar ROM was lower than that of the controls (Figures 5C, D). Our findings demonstrated that intra-spinal compensatory mechanisms may play a role in spinal ROM.

### 3.5 Assessment of osteogenic protein expression

**FIGURE 6:**
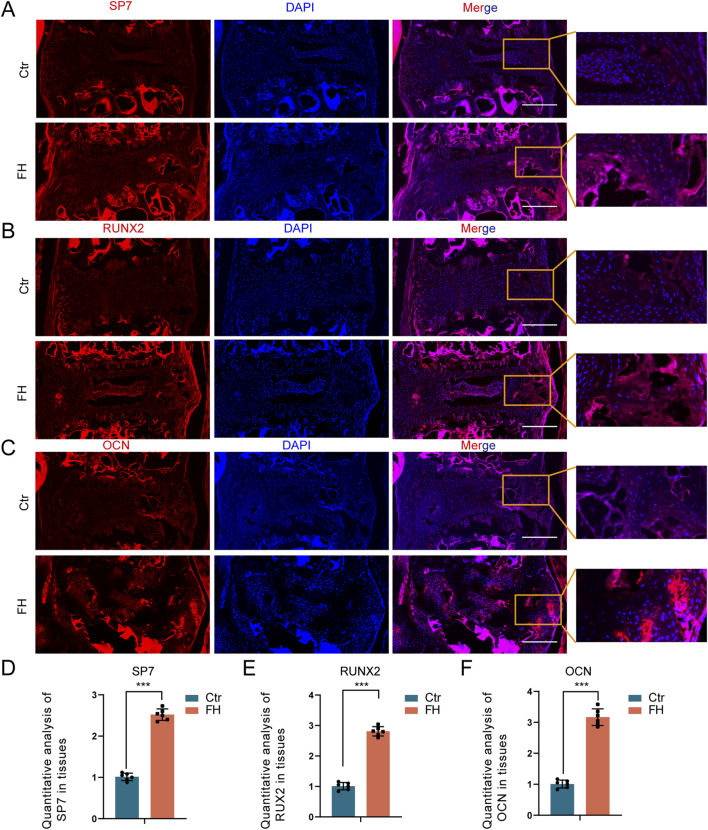
Effect of hypermobility on osteogenic protein expression. **(A–C)** IF staining of SP7,RUXN2 and OCN in PLL tissues. Scale bar = 250μm, n = 3. **(D–F)** IF staining quantification assay of SP7,RUXN2 and OCN, all data were presented as means ± SD, n = 6, *p < 0.05, **p < 0.01, ***p < 0.001.

We then examined the effect of hypermobility on osteogenic protein expression in the PLL tissue. Immunofluorescence (IF) staining showed increased expression of SP7, RUXN2 and OCN after 24 weeks of stimulation (Figures 6A–C). IF staining quantitative assay revealed that SP7, RUNX2, and OCN expression of the FH group was significantly higher than the controls (Figures 6D–F). These findings imply that hypermobility of the spine encouraged osteogenic protein expression in PLL tissue of *Enpp1*-deficient mice.

### 3.6 Activation of DLX5 and NOTCH signaling pathway

**FIGURE 7:**
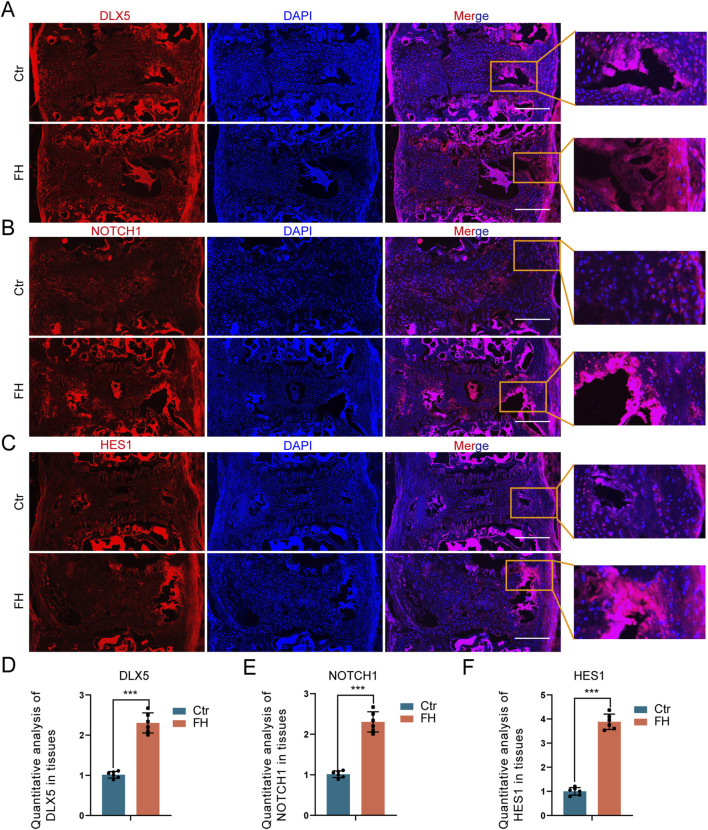
Effect of hypermobility on DLX5 and NOTCH signaling expression. **(A–C)** IF staining of DLX5, NOTCH1 and HES1 in PLL tissues. Scale bar = 250μm, n = 6. **(D–F)** IF staining quantification assay of DLX5, NOTCH1 and HES1, all data were presented as means ± SD, n = 6, *p < 0.05, **p < 0.01, ***p < 0.001.

We also investigated the influence of hypermobility on DLX5 and NOTCH signaling in the PLL tissue. IF staining showed increased expression of DLX5, NOTCH1 and HES1 after 24 weeks of stimulation (Figures 7A–C). The quantitative IF staining assay revealed that the expression of DLX5, NOTCH1, and HES1 was higher in the FH group than in the controls (Figures 7D–F). Our results suggest that spinal hypermobility promotes the production of DLX5 and NOTCH signaling in PLL tissue of *Enpp1*-deficient mice.

## 4 Discussion

OPLL is a common illness in Asia and surgery is the only treatment option for symptomatic OPLL (Xue et al., 2023). The underlying mechanism of OPLL is still unknown, however excessive movement of the spine has been identified as one of the major contributors in the course of the OPLL (Shi et al., 2019; Won et al., 2022). We previously constructed the mice with heterotopic ossification of the spine and micro-CT imaging revealed heterotopic ossification of the PLL (Zhu et al., 2023a). In this investigation, we found that spinal hypermobility increase ossification development of PLL in the *Enpp1*-deficient mice.

ENPP1 deficiency has also been linked to a progressive spinal stiffness condition known as Ossification of the Posterior Longitude Ligament (Mehta et al., 2012; Kotwal et al., 2020). Murine models of OPLL, produced by Japanese researchers based on phenotypic, have been shown to have loss of function mutations in *Enpp1* (a Gly568stop mutation) that cause the OPLL phenotype (A et al., 1998). Both environmental and genetic factors play a part in the development of OPLL. However, SNPs in *Enpp1* have been linked to OPLL, one of which is strongly linked to severe ossification and a younger age of onset (Koshizuka et al., 2002). In this study, we found that the ectopic ossification of spinal ligaments in mice by Micro-CT. OPLL is associated with spinal instability and mostly occurs in the segment with the greatest range of motion, while posterior cervical decompression promotes progression of ossification (Chen et al., 2018; Oshima et al., 2020).

Mechanical factor is known to be an important regulator of bone reconstruction, increasing the number of osteoblasts and the expression levels of osteogenic marker genes (Harter et al., 1995; Zhang et al., 2017). In this experiment we found that the ossification of the PLL was increased after 8 weeks of stimulation. Besides, the relative area of ossification in the spinal canal was larger in the hypermobility group than in the controls. In conclusion, these results suggest that abnormal hypermobility plays a key role in the progression of heterotopic ossification of ligaments.

A novel stimulation device consisting of a moving bed, immobilizers, strain gauges, etc. was designed to apply cyclic tensile strain to the rat caudal spinal ligaments (Tsukamoto et al., 2006). Besides, eccentric wheel, steel rod and V-beam can be used to exert stretch on the rats ligamentum flavum (Zhao et al., 2021). Although the above device allows for periodic flexion of the ligament, it still cannot fully simulate the active flexion of the human cervical spine. In addition, there is a risk of limb and ligament injury, epidermal infection and spinal cord injury. In addition, a new bipedal standing mouse model has been developed, but this model lacks imaging support and has few selected time points (Ao et al., 2019). The device we used in this study has the advantage of fully simulating the active flexion of the human cervical spine and its non-invasiveness, providing a suitable animal model for studying the progression of hypermobility in OPLL.

The compression of the spinal cordfigure lateral destruction of the white and gray matter and the formation of cystic cavities (Khodanovich et al., 2018; Ouellette et al., 2020). Several studies have shown that *Enpp1*-deficient mice is a suitable animal model for studying long-term mechanical compression of the spinal cord (Takeura et al., 2019; Ichikawa et al., 2021). Following 16 weeks of stimulation, both the BMS score and maximum angle of stay on the inclined plate for 5 s of hypermobility group were significantly lower than the controls. The effects of hypermobility on spinal cord symptoms manifest later than the growth in ossification. This is because early ossified areas have milder compression on the spinal cord, and other unaffected regions can compensate. However, in later stages, the necrotic portion of the spinal cord increases, and compensatory mechanisms become insufficient for normal motor activities *in vivo*.

Several studies have indicated that mechanical factors increases the expression of DLX5 in human bone marrow mesenchymal stem cells (MSCs) (Sugii et al., 2017; Papadogiannis et al., 2020). This upregulation of DLX5 is thought to be mediated through the activation of the PKA signaling pathway (Lee et al., 2018). Furthermore, DLX5 may enhance osteogenic differentiation of MSCs in combination with hyperactivity-responsive molecules such as YAP under mechanical stimulation (Lee et al., 2003; Holleville et al., 2007). NOTCH1 responds to mechanical stimulation and regulates endothelial cell proliferation, differentiation, apoptosis and differentiation (Mack et al., 2017; Bagheri et al., 2018; Man et al., 2018). Our latest study demonstrated the significance of DLX5 regulates the osteogenic differentiation of spinal ligaments cells derived from ossification of the posterior longitudinal ligament patients via NOTCH signaling under cyclic stretch (Tang et al., 2023). In this study, it showed that DLX5, NOTCH1 and HES1 expression were significantly increased in PLL after 24 weeks of stimulation in the FH group. In addition, our previous study showed that uniaxial cyclic stretch induced activation of the NOTCH signaling pathway to release NICD, which translocated from the cytoplasm to the nucleus to regulate the expression of DLX5 (Bagheri et al., 2018). In summary, the hypermobility of the spine promotes the expression of DLX5 and NOTCH signaling in PLL tissues of *Enpp1-deficient* mice.

The present study has some limitations. First, the device consisting of a water dripper and a flow valve to induce repeated neck flexion and extension motions in mice, the effect of water as an external factor on the progression of ligament ossification in mice could not be excluded. Second, we only used Micro-ct on mice, and it was difficult to detect subtle changes in the ligaments of *Enpp1*-deficient mice. We then needed more advanced and precise methods to quantify changes in heterotopic ossification of the spine in mice, such as 3D propagation phase contrast micro tomography (Zheng et al., 2018). Third, this experiment only verified the high expression levels of DLX5 and NOTCH signaling in hypermobility of the spine at tissue level, and further corroboration is needed by injecting shDLX5 lentivirus into the tail vein of mice. Fourth, the genetically deficient mice employed in this work exhibit ossification of ligament tissue throughout the body rather than only in the PLL, which may influence experimental results. Finally, the pathological sections in the HE staining exhibit detachment, the main reason for the detachment is that spinal tissue is relatively fragile and prone to detachment during the sectioning process. Although there are issues with the images, we believe they still provide valuable scientific information overall, and the analysis results are not significantly affected.

## 5 Conclusion

In this study we established an animal model to study the role of hypermobility of the spine in OPLL. Our results suggest that excessive movement of the spine promotes ossification progression in mice with heterotopic ossification of spinal ligaments, accelerates motor function reduction and reduces range of motion in the cervicothoracic and thoracolumbar spine. In addition, DLX5 and NOTCH signaling pathways may be involved in ossification progression induced by spinal hypermobility. This finding provides new insights to understand the pathogenesis of OPLL.

## Data Availability

The original contributions presented in the study are included in the article/Supplementary Material, further inquiries can be directed to the corresponding authors.

## Supplementary material

SUPPLEMENTARY FIGURE S1 Spinal hypermobility and gross spinal specimens in Enpp1-deficient mice. **(A)** The mice were “tiptoe walking” on the board and walking was recorded both before and after hypermobility. **(B)** Spinal hypermobility was applied to mice in the control, SH, and FH group in an upright container. **(C)** Typical mouse gross spinal specimens after various stimulation durations.
