# Supplementary figures and images for: Spinal hypermobility accelerates ossification in posterior longitudinal ligaments: insights from an in vivo mouse model
Source: Front Physiol. 2025 Mar 19;16:1561199. doi: 10.3389/fphys.2025.1561199 (PMC11962021; doi:10.3389/fphys.2025.1561199)

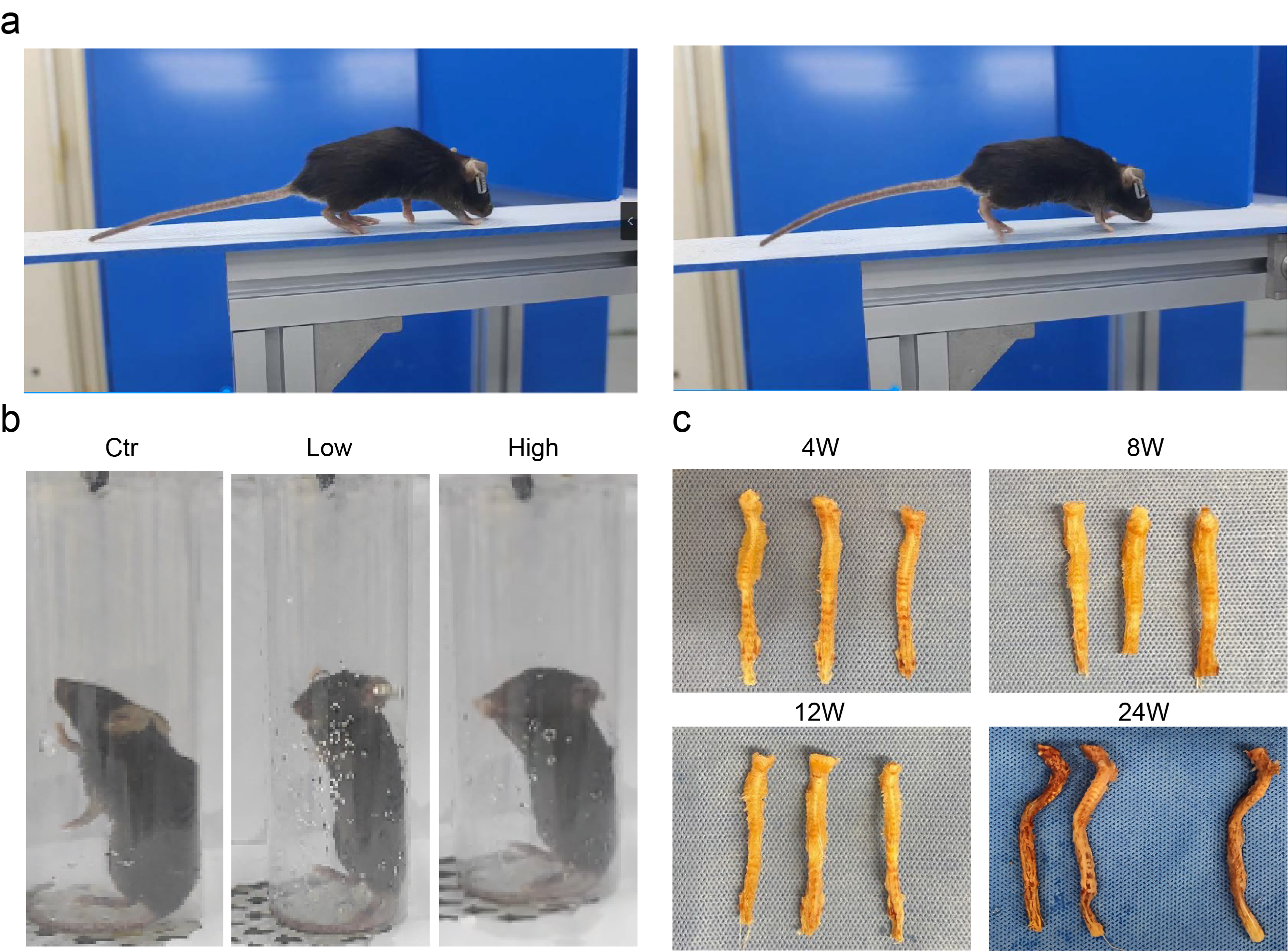

Supplement: Supplementary file 1 [file Image1.jpeg]
